# Di-μ-tricyanido-tetra­cyanidobis[hydro­tris­(pyrazoylborato)]tetra­methanol­diiron(III)iron(II) di­methanol disolvate

**DOI:** 10.1107/S1600536814001019

**Published:** 2014-01-22

**Authors:** Kuirun Zhang, Osamu Sato

**Affiliations:** a Institute for Materials Chemistry and Engineering, Kyushu University, 6-1, Kasuga-koen, Fukuoka 816-0811, Japan

## Abstract

In the title complex, [Fe^II^Fe^III^_2_(C_9_H_10_BN_6_)_2_(CN)_6_(CH_3_OH)_4_]·2CH_3_OH, two [Fe^III^(Tp)(CN)_3_]^−^ anions [Tp is hydro­tris­(pyrazoylborate)] are bridged by an [Fe^II^(MeOH)_4_]^2+^ cation, forming a centrosymmetric trinuclear unit. These units are connected *via* O—H⋯O and O—H⋯N hydrogen bonds involving the uncoordinated methanol solvent mol­ecules, forming a three-dimensional network.

## Related literature   

For the synthesis of bis­{tri­cyano­[hydro­tris­(pyrazoylborate)]ferrate(III)}, see Lescouëzec *et al.* (2002 ▶). For a related structure, see Kim *et al.* (2004 ▶).
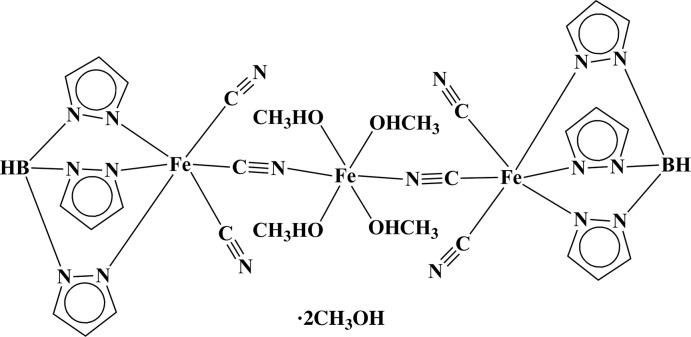


## Experimental   

### 

#### Crystal data  

[Fe_3_(C_9_H_10_BN_6_)_2_(CN)_6_(CH_4_O)_4_]·2CH_4_O
*M* *_r_* = 942.00
Monoclinic, 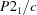

*a* = 9.261 (4) Å
*b* = 16.405 (7) Å
*c* = 14.331 (6) Å
β = 94.671 (2)°
*V* = 2169.9 (16) Å^3^
*Z* = 2
Mo *K*α radiation
μ = 1.05 mm^−1^
*T* = 123 K
0.30 × 0.20 × 0.10 mm

#### Data collection  

Rigaku Saturn70 diffractometer
Absorption correction: multi-scan (*SADABS*; Bruker, 2000 ▶) *T*_min_ = 0.743, *T*_max_ = 0.902
16575 measured reflections
4922 independent reflections
4557 reflections with *I* > 2σ(*I*)
*R*_int_ = 0.097

#### Refinement  

*R*[*F* ^2^ > 2σ(*F* ^2^)] = 0.064
*wR*(*F* ^2^) = 0.167
*S* = 1.13
4922 reflections
269 parameters
H-atom parameters constrained
Δρ_max_ = 0.95 e Å^−3^
Δρ_min_ = −0.82 e Å^−3^

### 

Data collection: *CrystalClear* (Rigaku, 2008 ▶); cell refinement: *CrystalClear*; data reduction: *CrystalClear*; program(s) used to solve structure: *SHELXS97* (Sheldrick, 2008 ▶); program(s) used to refine structure: *SHELXL97* (Sheldrick, 2008 ▶); molecular graphics: *XP* in *SHELXTL* (Sheldrick, 2008 ▶); software used to prepare material for publication: *publCIF* (Westrip, 2010 ▶).

## Supplementary Material

Crystal structure: contains datablock(s) I. DOI: 10.1107/S1600536814001019/pk2510sup1.cif

Structure factors: contains datablock(s) I. DOI: 10.1107/S1600536814001019/pk2510Isup2.hkl

CCDC reference:

Additional supporting information:  crystallographic information; 3D view; checkCIF report

## Figures and Tables

**Table 1:** Hydrogen-bond geometry (Å, °)

*D*—H⋯*A*	*D*—H	H⋯*A*	*D*⋯*A*	*D*—H⋯*A*
O1—H1O⋯O3	0.93	1.75	2.645 (4)	161
O2—H2O⋯N7^i^	0.83	1.97	2.769 (4)	161
O3—H3O⋯N9^ii^	0.85	1.97	2.815 (4)	172
O1—H1O⋯O3	0.93	1.75	2.645 (4)	161
O2—H2O⋯N7^i^	0.83	1.97	2.769 (4)	161
C8—H8⋯N9^iii^	0.95	2.62	3.523 (5)	158
O3—H3O⋯N9^ii^	0.85	1.97	2.815 (4)	172

Symmetry codes: (i) 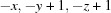; (ii) 
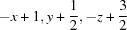
; (iii) 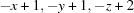.

**Fig. 1.**
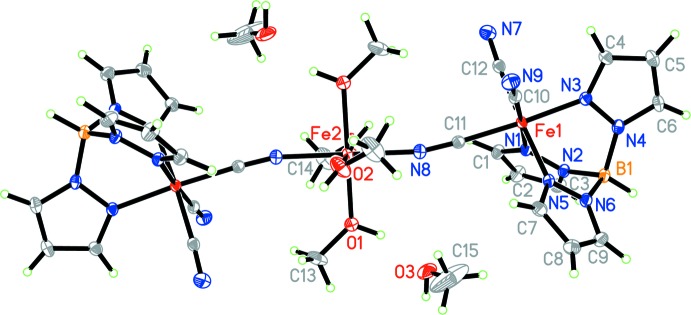
The displacement ellipsoid plot (50% probability level) of the title compound. Unlabelled atoms are related to their labelled counterparts by the symmetry operation (–x + 1, -y + 1, -z + 1).

**Fig. 2.**
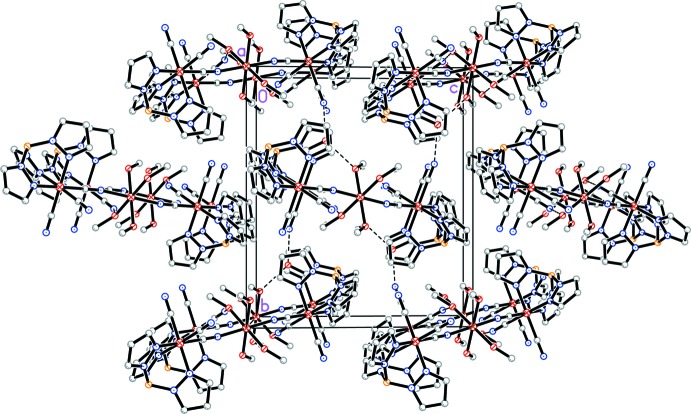
The packing diagram of the title compound viewed along the crystallographic a–axis. H atoms are omitted to enhance clarity.

## supplementary crystallographic information

### 1. Introduction

A trinuclear cyanide bridged complex was synthesized by slow evaporation. In this compound, the central iron(II) ion is coordinated by two nitro­gen atoms from cyanide bridging of [Fe^III^(Tp)(CN)3]^-^ (Tp = hydro­tris­(pyrazoylborate) with *trans* geometry, and four oxygen atoms from coordinated methanol molecules. While, the Fe^III^ in the [Fe^III^(Tp)(CN)3]^-^ part is coordinated by three nitro­gen atoms from Tp^-^ and three carbon atoms from cyanide. The Fe1—N—C—Fe2 structure unit is almost linear with Fe1—C—N and Fe2—N—C angles of 176.1 (3)° and 168.2 (3)°, respectively. Two types of hydrogen bonds O1—H10···O3 and O3—H30···N9 are present between the uncoordinated methanol molecules and [Fe^III^(Tp)(CN)_3_]_2_[Fe^II^(MeOH)_4_] units, linking the trinuclear units into a three-dimensional supra-molecule. The carbon atom of the uncoordinated methanol molecules is probably disordered. An isostructural compound [Fe^III^(Tp)(CN)_3_]_2_[Mn^II^(MeOH)_4_] and two methanol molecules was reported previously (Kim *et al.*, 2004).

### 2. Experimental

Tetra-n-butyl­ammonium bis-[tri­cyano-hydro­tris­(pyrazoylborate)-ferrate(III)] (0.589g,0.1mmol) and ferrous perchlorate hydrated (0.018g. 0.05 mmol) in 10 ml methanol were reacted for 30 min at room temperature. Slow evaporation of the filtrate gave red crystals.

### 2.1. Refinement

Carbon-bound H-atoms of pyrazole were placed in calculated positions (C—H 0.95 Å) and were included in the refinement in the riding model approximation, with U_iso_(H) set to 1.2U_eq_(C). The carbon-bound H-atoms of methanol, O-bound H-atoms and B-bound H-atoms were located in a difference Fourier map, and were refined with a distance restraints of C—H, O—H and B—H 0.98±0.01 Å, 0.82±0.1 Å, 1.12 Å, respectively; with U_iso_(H) set to 1.2U_eq_(N).

Crystal data, data collection and structure refinement details are summarized in Table 1.

### Crystal data

**Table:**

[Fe_3_(C_9_H_10_BN_6_)_2_(CN)_6_(CH_4_O)_4_]·2CH_4_O	*F*(000) = 972
*M**_r_* = 942.00	*D*_x_ = 1.442 Mg m^−^^3^
Monoclinic, *P*2_1_/*c*	Mo *K*α radiation, λ = 0.7107 Å
Hall symbol: -P 2ybc	Cell parameters from 4554 reflections
*a* = 9.261 (4) Å	θ = 3.1–27.5°
*b* = 16.405 (7) Å	µ = 1.05 mm^−^^1^
*c* = 14.331 (6) Å	*T* = 123 K
β = 94.671 (2)°	Block, red
*V* = 2169.9 (16) Å^3^	0.30 × 0.20 × 0.10 mm
*Z* = 2	

### Data collection

**Table:**

Rigaku Saturn70 diffractometer	4922 independent reflections
Radiation source: fine-focus sealed tube	4557 reflections with *I* > 2σ(*I*)
Graphite monochromator	*R*_int_ = 0.097
Detector resolution: 7.314 pixels mm^-1^	θ_max_ = 27.5°, θ_min_ = 3.1°
ω scans	*h* = −12→11
Absorption correction: multi-scan (*SADABS*; Bruker, 2000)	*k* = −21→21
*T*_min_ = 0.743, *T*_max_ = 0.902	*l* = −18→16
16575 measured reflections	

### Refinement

**Table:**

Refinement on *F*^2^	Primary atom site location: structure-invariant direct methods
Least-squares matrix: full	Secondary atom site location: difference Fourier map
*R*[*F*^2^ > 2σ(*F*^2^)] = 0.064	Hydrogen site location: inferred from neighbouring sites
*wR*(*F*^2^) = 0.167	H-atom parameters constrained
*S* = 1.13	*w* = 1/[σ^2^(*F*_o_^2^) + (0.0721*P*)^2^ + 4.4189*P*] where *P* = (*F*_o_^2^ + 2*F*_c_^2^)/3
4922 reflections	(Δ/σ)_max_ = 0.001
269 parameters	Δρ_max_ = 0.95 e Å^−^^3^
0 restraints	Δρ_min_ = −0.82 e Å^−^^3^

### Special details

**Table:**

Geometry. All e.s.d.'s (except the e.s.d. in the dihedral angle between two l.s. planes) are estimated using the full covariance matrix. The cell e.s.d.'s are taken into account individually in the estimation of e.s.d.'s in distances, angles and torsion angles; correlations between e.s.d.'s in cell parameters are only used when they are defined by crystal symmetry. An approximate (isotropic) treatment of cell e.s.d.'s is used for estimating e.s.d.'s involving l.s. planes.
Refinement. Refinement of *F*^2^ against ALL reflections. The weighted *R*-factor *wR* and goodness of fit *S* are based on *F*^2^, conventional *R*-factors *R* are based on *F*, with *F* set to zero for negative *F*^2^. The threshold expression of *F*^2^ > σ(*F*^2^) is used only for calculating *R*-factors(gt) *etc*. and is not relevant to the choice of reflections for refinement. *R*-factors based on *F*^2^ are statistically about twice as large as those based on *F*, and *R*- factors based on ALL data will be even larger.

### Fractional atomic coordinates and isotropic or equivalent isotropic displacement parameters (Å^2^)

**Table:**

	*x*	*y*	*z*	*U*_iso_*/*U*_eq_	
Fe1	0.16211 (4)	0.53724 (3)	0.76431 (3)	0.01108 (15)	
Fe2	0.5000	0.5000	0.5000	0.01447 (18)	
O1	0.6195 (3)	0.61004 (16)	0.52139 (18)	0.0259 (6)	
H1O	0.5856	0.6453	0.5654	0.031*	
O2	0.3481 (3)	0.57099 (18)	0.41549 (19)	0.0295 (6)	
H2O	0.2586	0.5732	0.4119	0.035*	
O3	0.5843 (4)	0.71803 (18)	0.6547 (2)	0.0424 (8)	
H3O	0.6238	0.7647	0.6527	0.051*	
N1	0.1053 (3)	0.64652 (16)	0.71911 (18)	0.0132 (5)	
N2	0.0729 (3)	0.70462 (16)	0.78192 (18)	0.0139 (5)	
N3	0.0040 (3)	0.54145 (16)	0.84969 (18)	0.0136 (5)	
N4	−0.0165 (3)	0.61102 (16)	0.89905 (18)	0.0146 (5)	
N5	0.2907 (3)	0.58953 (16)	0.86392 (18)	0.0133 (5)	
N6	0.2409 (3)	0.65368 (16)	0.91250 (18)	0.0151 (5)	
N7	−0.0531 (3)	0.45434 (18)	0.6197 (2)	0.0223 (6)	
N8	0.3854 (3)	0.52601 (18)	0.6178 (2)	0.0192 (6)	
N9	0.2724 (3)	0.36947 (17)	0.8348 (2)	0.0210 (6)	
C1	0.0913 (4)	0.6807 (2)	0.6342 (2)	0.0180 (6)	
H1	0.1081	0.6539	0.5774	0.022*	
C2	0.0484 (4)	0.7615 (2)	0.6417 (2)	0.0218 (7)	
H2	0.0302	0.7998	0.5924	0.026*	
C3	0.0379 (4)	0.77434 (19)	0.7362 (2)	0.0191 (7)	
H3	0.0107	0.8239	0.7642	0.023*	
C4	−0.0966 (4)	0.4884 (2)	0.8710 (2)	0.0169 (6)	
H4	−0.1068	0.4348	0.8462	0.020*	
C5	−0.1852 (4)	0.5225 (2)	0.9348 (2)	0.0199 (7)	
H5	−0.2653	0.4978	0.9612	0.024*	
C6	−0.1310 (3)	0.5999 (2)	0.9513 (2)	0.0180 (6)	
H6	−0.1676	0.6390	0.9922	0.022*	
C7	0.4266 (3)	0.5739 (2)	0.9010 (2)	0.0179 (6)	
H7	0.4877	0.5322	0.8805	0.021*	
C8	0.4640 (4)	0.6281 (2)	0.9736 (2)	0.0225 (7)	
H8	0.5528	0.6307	1.0116	0.027*	
C9	0.3438 (4)	0.6775 (2)	0.9786 (2)	0.0197 (7)	
H9	0.3353	0.7210	1.0216	0.024*	
C10	0.2287 (3)	0.4314 (2)	0.8080 (2)	0.0150 (6)	
C11	0.3056 (3)	0.53102 (18)	0.6750 (2)	0.0138 (6)	
C12	0.0301 (3)	0.48514 (19)	0.6716 (2)	0.0149 (6)	
C13	0.7555 (5)	0.6320 (3)	0.4880 (4)	0.0401 (11)	
H13A	0.7890	0.6835	0.5168	0.048*	
H13B	0.7438	0.6384	0.4198	0.048*	
H13C	0.8267	0.5891	0.5043	0.048*	
C14	0.3792 (5)	0.6167 (3)	0.3344 (3)	0.0386 (10)	
H14A	0.3525	0.5845	0.2780	0.046*	
H14B	0.4830	0.6293	0.3376	0.046*	
H14C	0.3235	0.6676	0.3321	0.046*	
C15	0.6291 (13)	0.6822 (4)	0.7417 (4)	0.123 (5)	
H15A	0.7292	0.6981	0.7604	0.148*	
H15B	0.6232	0.6227	0.7362	0.148*	
H15C	0.5658	0.7007	0.7891	0.148*	
B1	0.0838 (4)	0.6841 (2)	0.8869 (2)	0.0150 (7)	
H10	0.0501	0.7378	0.9281	0.018*	

### Atomic displacement parameters (Å^2^)

**Table:**

	*U*^11^	*U*^22^	*U*^33^	*U*^12^	*U*^13^	*U*^23^
Fe1	0.0115 (2)	0.0095 (2)	0.0126 (2)	0.00115 (15)	0.00292 (16)	0.00043 (15)
Fe2	0.0125 (3)	0.0171 (3)	0.0143 (3)	−0.0025 (2)	0.0040 (2)	−0.0033 (2)
O1	0.0240 (13)	0.0255 (13)	0.0297 (14)	−0.0087 (11)	0.0112 (10)	−0.0113 (11)
O2	0.0152 (12)	0.0420 (16)	0.0312 (14)	0.0014 (11)	0.0002 (10)	0.0111 (12)
O3	0.065 (2)	0.0274 (15)	0.0368 (16)	−0.0274 (15)	0.0179 (15)	−0.0142 (12)
N1	0.0154 (12)	0.0117 (12)	0.0127 (12)	0.0005 (10)	0.0030 (9)	0.0004 (9)
N2	0.0175 (12)	0.0117 (12)	0.0126 (12)	0.0018 (10)	0.0022 (9)	−0.0003 (10)
N3	0.0144 (13)	0.0112 (12)	0.0156 (13)	0.0019 (10)	0.0033 (10)	−0.0001 (9)
N4	0.0146 (12)	0.0141 (13)	0.0154 (12)	0.0045 (10)	0.0028 (10)	−0.0012 (10)
N5	0.0126 (12)	0.0145 (12)	0.0131 (12)	0.0008 (10)	0.0028 (9)	−0.0008 (10)
N6	0.0177 (13)	0.0135 (12)	0.0146 (12)	−0.0001 (10)	0.0043 (10)	−0.0017 (10)
N7	0.0203 (14)	0.0233 (15)	0.0231 (15)	−0.0024 (12)	0.0006 (12)	0.0013 (12)
N8	0.0192 (14)	0.0198 (14)	0.0194 (14)	−0.0016 (11)	0.0059 (11)	−0.0040 (11)
N9	0.0241 (15)	0.0135 (13)	0.0252 (15)	0.0025 (11)	0.0001 (11)	0.0003 (11)
C1	0.0218 (16)	0.0173 (15)	0.0151 (15)	0.0010 (13)	0.0018 (12)	0.0008 (12)
C2	0.0341 (19)	0.0143 (15)	0.0167 (15)	0.0032 (14)	−0.0004 (14)	0.0049 (12)
C3	0.0255 (17)	0.0099 (14)	0.0217 (16)	0.0019 (13)	0.0001 (13)	0.0013 (12)
C4	0.0156 (15)	0.0158 (15)	0.0195 (15)	−0.0003 (12)	0.0033 (12)	0.0024 (12)
C5	0.0157 (15)	0.0239 (17)	0.0210 (16)	0.0028 (13)	0.0068 (12)	0.0039 (13)
C6	0.0158 (15)	0.0235 (16)	0.0154 (14)	0.0034 (13)	0.0052 (11)	0.0015 (12)
C7	0.0132 (14)	0.0202 (16)	0.0202 (15)	0.0011 (12)	0.0010 (12)	0.0011 (13)
C8	0.0169 (15)	0.0267 (18)	0.0234 (17)	−0.0024 (14)	−0.0011 (13)	−0.0015 (14)
C9	0.0225 (16)	0.0202 (16)	0.0162 (15)	−0.0027 (13)	0.0009 (12)	−0.0025 (12)
C10	0.0140 (14)	0.0170 (15)	0.0140 (14)	0.0013 (12)	0.0012 (11)	−0.0017 (11)
C11	0.0148 (14)	0.0107 (14)	0.0160 (15)	0.0002 (11)	0.0019 (11)	0.0001 (11)
C12	0.0139 (14)	0.0136 (14)	0.0175 (15)	0.0023 (12)	0.0040 (11)	0.0008 (12)
C13	0.027 (2)	0.037 (2)	0.057 (3)	−0.0175 (18)	0.0123 (19)	−0.009 (2)
C14	0.027 (2)	0.049 (3)	0.041 (2)	0.0047 (19)	0.0040 (17)	0.018 (2)
C15	0.272 (13)	0.069 (4)	0.027 (3)	−0.107 (7)	0.007 (5)	0.001 (3)
B1	0.0174 (16)	0.0145 (16)	0.0134 (15)	0.0013 (13)	0.0030 (12)	−0.0008 (12)

### Geometric parameters (Å, º)

**Table:**

Fe1—C11	1.922 (3)	N6—B1	1.554 (4)
Fe1—C10	1.930 (3)	N7—C12	1.144 (5)
Fe1—C12	1.930 (3)	N8—C11	1.150 (4)
Fe1—N1	1.963 (3)	N9—C10	1.148 (4)
Fe1—N5	1.979 (3)	C1—C2	1.391 (5)
Fe1—N3	1.984 (3)	C1—H1	0.9500
Fe2—N8^i^	2.109 (3)	C2—C3	1.382 (5)
Fe2—N8	2.109 (3)	C2—H2	0.9500
Fe2—O1^i^	2.127 (3)	C3—H3	0.9500
Fe2—O1	2.127 (3)	C4—C5	1.395 (5)
Fe2—O2	2.127 (3)	C4—H4	0.9500
Fe2—O2^i^	2.127 (3)	C5—C6	1.379 (5)
O1—C13	1.429 (5)	C5—H5	0.9500
O1—H1O	0.9286	C6—H6	0.9500
O2—C14	1.432 (5)	C7—C8	1.390 (5)
O2—H2O	0.8267	C7—H7	0.9500
O3—C15	1.410 (8)	C8—C9	1.383 (5)
O3—H3O	0.8500	C8—H8	0.9500
N1—C1	1.336 (4)	C9—H9	0.9500
N1—N2	1.361 (4)	C13—H13A	0.9800
N2—C3	1.344 (4)	C13—H13B	0.9800
N2—B1	1.537 (4)	C13—H13C	0.9800
N3—C4	1.329 (4)	C14—H14A	0.9800
N3—N4	1.364 (4)	C14—H14B	0.9800
N4—C6	1.359 (4)	C14—H14C	0.9800
N4—B1	1.535 (5)	C15—H15A	0.9800
N5—C7	1.350 (4)	C15—H15B	0.9800
N5—N6	1.363 (4)	C15—H15C	0.9800
N6—C9	1.345 (4)	B1—H10	1.1191
			
C11—Fe1—C10	87.03 (13)	C11—N8—Fe2	168.2 (3)
C11—Fe1—C12	87.23 (14)	N1—C1—C2	109.7 (3)
C10—Fe1—C12	89.59 (13)	N1—C1—H1	125.1
C11—Fe1—N1	90.58 (12)	C2—C1—H1	125.1
C10—Fe1—N1	176.91 (12)	C3—C2—C1	105.3 (3)
C12—Fe1—N1	92.27 (12)	C3—C2—H2	127.4
C11—Fe1—N5	95.46 (12)	C1—C2—H2	127.4
C10—Fe1—N5	89.89 (12)	N2—C3—C2	108.3 (3)
C12—Fe1—N5	177.23 (12)	N2—C3—H3	125.8
N1—Fe1—N5	88.37 (11)	C2—C3—H3	125.8
C11—Fe1—N3	176.14 (12)	N3—C4—C5	110.4 (3)
C10—Fe1—N3	93.65 (12)	N3—C4—H4	124.8
C12—Fe1—N3	88.97 (13)	C5—C4—H4	124.8
N1—Fe1—N3	88.87 (11)	C6—C5—C4	104.9 (3)
N5—Fe1—N3	88.34 (11)	C6—C5—H5	127.6
N8^i^—Fe2—N8	179.999 (1)	C4—C5—H5	127.6
N8^i^—Fe2—O1^i^	90.17 (10)	N4—C6—C5	108.6 (3)
N8—Fe2—O1^i^	89.83 (10)	N4—C6—H6	125.7
N8^i^—Fe2—O1	89.83 (10)	C5—C6—H6	125.7
N8—Fe2—O1	90.17 (10)	N5—C7—C8	109.9 (3)
O1^i^—Fe2—O1	179.998 (1)	N5—C7—H7	125.0
N8^i^—Fe2—O2	90.45 (11)	C8—C7—H7	125.0
N8—Fe2—O2	89.55 (11)	C9—C8—C7	105.1 (3)
O1^i^—Fe2—O2	94.03 (11)	C9—C8—H8	127.4
O1—Fe2—O2	85.97 (11)	C7—C8—H8	127.4
N8^i^—Fe2—O2^i^	89.55 (11)	N6—C9—C8	108.7 (3)
N8—Fe2—O2^i^	90.45 (11)	N6—C9—H9	125.7
O1^i^—Fe2—O2^i^	85.97 (11)	C8—C9—H9	125.7
O1—Fe2—O2^i^	94.03 (11)	N9—C10—Fe1	177.9 (3)
O2—Fe2—O2^i^	180.000 (1)	N8—C11—Fe1	176.1 (3)
C13—O1—Fe2	128.9 (2)	N7—C12—Fe1	176.7 (3)
C13—O1—H1O	115.3	O1—C13—H13A	109.5
Fe2—O1—H1O	115.2	O1—C13—H13B	109.5
C14—O2—Fe2	125.5 (2)	H13A—C13—H13B	109.5
C14—O2—H2O	101.2	O1—C13—H13C	109.5
Fe2—O2—H2O	132.2	H13A—C13—H13C	109.5
C15—O3—H3O	108.1	H13B—C13—H13C	109.5
C1—N1—N2	107.3 (3)	O2—C14—H14A	109.5
C1—N1—Fe1	133.4 (2)	O2—C14—H14B	109.5
N2—N1—Fe1	119.27 (19)	H14A—C14—H14B	109.5
C3—N2—N1	109.3 (3)	O2—C14—H14C	109.5
C3—N2—B1	131.1 (3)	H14A—C14—H14C	109.5
N1—N2—B1	119.6 (2)	H14B—C14—H14C	109.5
C4—N3—N4	107.4 (3)	O3—C15—H15A	109.5
C4—N3—Fe1	133.3 (2)	O3—C15—H15B	109.5
N4—N3—Fe1	119.4 (2)	H15A—C15—H15B	109.5
C6—N4—N3	108.8 (3)	O3—C15—H15C	109.5
C6—N4—B1	132.3 (3)	H15A—C15—H15C	109.5
N3—N4—B1	118.9 (3)	H15B—C15—H15C	109.5
C7—N5—N6	106.8 (3)	N4—B1—N2	106.8 (3)
C7—N5—Fe1	133.6 (2)	N4—B1—N6	106.7 (3)
N6—N5—Fe1	119.6 (2)	N2—B1—N6	106.7 (2)
C9—N6—N5	109.5 (3)	N4—B1—H10	111.0
C9—N6—B1	132.0 (3)	N2—B1—H10	110.1
N5—N6—B1	118.5 (2)	N6—B1—H10	115.1
			
N8^i^—Fe2—O1—C13	−20.3 (4)	N8^i^—Fe2—N8—C11	164 (4)
N8—Fe2—O1—C13	159.7 (4)	O1^i^—Fe2—N8—C11	−20.8 (14)
O1^i^—Fe2—O1—C13	−88 (7)	O1—Fe2—N8—C11	159.2 (14)
O2—Fe2—O1—C13	−110.8 (4)	O2—Fe2—N8—C11	73.2 (14)
O2^i^—Fe2—O1—C13	69.2 (4)	O2^i^—Fe2—N8—C11	−106.8 (14)
N8^i^—Fe2—O2—C14	−31.2 (3)	N2—N1—C1—C2	−0.4 (4)
N8—Fe2—O2—C14	148.8 (3)	Fe1—N1—C1—C2	−179.5 (2)
O1^i^—Fe2—O2—C14	−121.4 (3)	N1—C1—C2—C3	0.3 (4)
O1—Fe2—O2—C14	58.5 (3)	N1—N2—C3—C2	−0.3 (4)
O2^i^—Fe2—O2—C14	12 (53)	B1—N2—C3—C2	178.1 (3)
C11—Fe1—N1—C1	38.3 (3)	C1—C2—C3—N2	0.0 (4)
C10—Fe1—N1—C1	78 (2)	N4—N3—C4—C5	0.0 (4)
C12—Fe1—N1—C1	−48.9 (3)	Fe1—N3—C4—C5	−178.8 (2)
N5—Fe1—N1—C1	133.8 (3)	N3—C4—C5—C6	−0.3 (4)
N3—Fe1—N1—C1	−137.9 (3)	N3—N4—C6—C5	−0.4 (4)
C11—Fe1—N1—N2	−140.6 (2)	B1—N4—C6—C5	178.7 (3)
C10—Fe1—N1—N2	−101 (2)	C4—C5—C6—N4	0.4 (4)
C12—Fe1—N1—N2	132.1 (2)	N6—N5—C7—C8	0.1 (4)
N5—Fe1—N1—N2	−45.2 (2)	Fe1—N5—C7—C8	−177.2 (2)
N3—Fe1—N1—N2	43.2 (2)	N5—C7—C8—C9	−0.1 (4)
C1—N1—N2—C3	0.4 (4)	N5—N6—C9—C8	0.0 (4)
Fe1—N1—N2—C3	179.6 (2)	B1—N6—C9—C8	177.9 (3)
C1—N1—N2—B1	−178.2 (3)	C7—C8—C9—N6	0.0 (4)
Fe1—N1—N2—B1	1.0 (4)	C11—Fe1—C10—N9	63 (8)
C11—Fe1—N3—C4	53.1 (19)	C12—Fe1—C10—N9	150 (8)
C10—Fe1—N3—C4	−47.0 (3)	N1—Fe1—C10—N9	23 (10)
C12—Fe1—N3—C4	42.5 (3)	N5—Fe1—C10—N9	−33 (8)
N1—Fe1—N3—C4	134.8 (3)	N3—Fe1—C10—N9	−121 (8)
N5—Fe1—N3—C4	−136.8 (3)	Fe2—N8—C11—Fe1	−25 (6)
C11—Fe1—N3—N4	−125.6 (17)	C10—Fe1—C11—N8	100 (4)
C10—Fe1—N3—N4	134.3 (2)	C12—Fe1—C11—N8	10 (4)
C12—Fe1—N3—N4	−136.2 (2)	N1—Fe1—C11—N8	−82 (4)
N1—Fe1—N3—N4	−43.9 (2)	N5—Fe1—C11—N8	−170 (4)
N5—Fe1—N3—N4	44.5 (2)	N3—Fe1—C11—N8	0 (6)
C4—N3—N4—C6	0.2 (3)	C11—Fe1—C12—N7	179 (100)
Fe1—N3—N4—C6	179.2 (2)	C10—Fe1—C12—N7	92 (5)
C4—N3—N4—B1	−179.1 (3)	N1—Fe1—C12—N7	−91 (5)
Fe1—N3—N4—B1	−0.1 (4)	N5—Fe1—C12—N7	12 (7)
C11—Fe1—N5—C7	−47.6 (3)	N3—Fe1—C12—N7	−2 (5)
C10—Fe1—N5—C7	39.4 (3)	C6—N4—B1—N2	−122.6 (3)
C12—Fe1—N5—C7	119 (2)	N3—N4—B1—N2	56.4 (3)
N1—Fe1—N5—C7	−138.0 (3)	C6—N4—B1—N6	123.6 (3)
N3—Fe1—N5—C7	133.0 (3)	N3—N4—B1—N6	−57.4 (3)
C11—Fe1—N5—N6	135.3 (2)	C3—N2—B1—N4	124.2 (4)
C10—Fe1—N5—N6	−137.6 (2)	N1—N2—B1—N4	−57.6 (3)
C12—Fe1—N5—N6	−58 (3)	C3—N2—B1—N6	−122.0 (4)
N1—Fe1—N5—N6	44.9 (2)	N1—N2—B1—N6	56.2 (3)
N3—Fe1—N5—N6	−44.0 (2)	C9—N6—B1—N4	−119.9 (4)
C7—N5—N6—C9	−0.1 (3)	N5—N6—B1—N4	57.8 (3)
Fe1—N5—N6—C9	177.7 (2)	C9—N6—B1—N2	126.1 (3)
C7—N5—N6—B1	−178.3 (3)	N5—N6—B1—N2	−56.1 (3)
Fe1—N5—N6—B1	−0.5 (3)		

Symmetry code: (i) −*x*+1, −*y*+1, −*z*+1.

### Hydrogen-bond geometry (Å, º)

**Table:**

*D*—H···*A*	*D*—H	H···*A*	*D*···*A*	*D*—H···*A*
O1—H1*O*···O3	0.93	1.75	2.645 (4)	161
O2—H2*O*···N7^ii^	0.83	1.97	2.769 (4)	161
O3—H3*O*···N9^iii^	0.85	1.97	2.815 (4)	172
O1—H1*O*···O3	0.93	1.75	2.645 (4)	161
O2—H2*O*···N7^ii^	0.83	1.97	2.769 (4)	161
C8—H8···N9^iv^	0.95	2.62	3.523 (5)	158
O3—H3*O*···N9^iii^	0.85	1.97	2.815 (4)	172

Symmetry codes: (ii) −*x*, −*y*+1, −*z*+1; (iii) −*x*+1, *y*+1/2, −*z*+3/2; (iv) −*x*+1, −*y*+1, −*z*+2.
